# Exploratory study of “real world” implementation of a clinical poverty tool in diverse family medicine and pediatric care settings

**DOI:** 10.1186/s12939-019-1085-0

**Published:** 2019-12-23

**Authors:** Eva Purkey, Imaan Bayoumi, Helen Coo, Allison Maier, Andrew D. Pinto, Bisola Olomola, Christina Klassen, Shannon French, Michael Flavin

**Affiliations:** 1 Department of Family Medicine, Queen’s University, 220 Bagot street, Kingston, Ontario K7L 5E9 Canada; 2 Department of Pediatrics, Queen’s University, Ontario, Canada; 3 Kingston, Frontenac and Lennox & Addington Public Health Unit, Kingston, Ontario Canada; 4 Department of Family and Community Medicine, Faculty of Medicine, University of Toronto, Dalla Lana School of Public Health, University of Toronto, The Upstream Lab, Centre for Urban Health Solutions, Li Ka Shing Knowledge Institute, St. Michael’s Hospital, Ontario, Canada; 5 Queen’s University, Ontario, Canada

**Keywords:** Poverty, Social determinants of health, Screening, Health care

## Abstract

**Background:**

Poverty is associated with increased morbidity related to multiple child and adult health conditions and increased risk of premature death. Despite robust evidence linking income and health, and some recommendations for universal screening, poverty screening is not routinely conducted in clinical care.

**Methods:**

We conducted an exploratory study of implementing universal poverty screening and intervention in family medicine and a range of pediatric care settings (primary through tertiary). After attending a training session, health care providers (HCPs) were instructed to perform universal screening using a clinical poverty tool with the question “Do you ever have difficulty making ends meet at the end of the month?” for the three-month implementation period. HCPs tracked the number of patients screened and a convenience sample of their patients were surveyed regarding the acceptability of being screened for poverty in a healthcare setting. HCPs participated in semi-structured focus groups to explore barriers to and facilitators of universal implementation of the tool.

**Results:**

Twenty-two HCPs (10 pediatricians, 9 family physicians, 3 nurse practitioners) participated and 150 patients completed surveys. Eighteen HCPs participated in focus groups. Despite the self-described motivation of the HCPs, screening rates were low (9% according to self-reported numbers). The majority of patients either supported (72%) or were neutral (22%) about the appropriateness of HCPs screening for and intervening on poverty. HCPs viewed poverty as relevant to clinical care but identified time constraints, physician discomfort, lack of expertise and habitual factors as barriers to implementation of universal screening.

**Conclusions:**

Poverty screening is important and acceptable to clinicians and patients. However, multiple barriers need to be addressed to allow for successful implementation of poverty screening and intervention in health care settings.

## Background

In 2017 about 10% of Candians were classified as low income [1] and a UNICEF report from that same year ranked Canada 32nd of 41 high income countries in child poverty [2]. Income is a powerful driver of health [3, 4]. Lower income is associated with a range of adverse health outcomes including shorter life expectancy, suicide, diabetes mellitus, myocardial infarction [3], and higher health care utilization [5]. Child poverty is associated with higher infant and child mortality and greater morbidity related to injury, asthma, and developmental delay [6–10]. The well-established link between income and health has increasingly led to calls by Canadian professional associations such as the College of Family Physicians of Canada [11] and the Canadian Medical Association [12, 13] to address poverty as a health issue.

Primary health care in Ontario, Canada’s most populous province, is delivered through a variety of practice models including physician only and team based models with blended capitation, salary and fee-for-service remuneration (further described below) [14]. Most primary care for children is provided by family physicians, but pediatricians also provide a substantial amount of primary care to children in Ontario, particularly in urban areas [15]. The Ontario College of Family Physicians recommends a clinical poverty tool [16, 17] to screen all patients for poverty using the question “Do you ever have difficulty making ends meet at the end of the month?” This question has a sensitivity of 98% [18] and a specificity of 40% [19] to identify people living below the poverty line. Health care providers (HCPs) are advised to use the response to the screening question to adjust health risk and to assist patients in accessing resources and benefits for which they are eligible. Likewise, the Canadian Pediatric Society has included recommendations related to screening for poverty within evidence informed guides such as the Rourke Baby Record [20] and the Grieg Health Record for children and adolescents [21]. Implementation of such practice level improvements is not supported by any province-wide initiatives in Ontario and little is known about the feasibility and acceptability of implementing clinical poverty tools within Ontario primary care pediatric and family medicine practices.

An additional consideration is whether poverty screening and intervention should be implemented at other levels of care [22]. Approximately 10% of Ontarians do not have a regular health care provider (e.g., family doctor) [23]. Given this, and Canada’s high child poverty rate relative to other developed countries, we aimed to evaluate the implementation of the Ontario College of Family Physicians’ clinical poverty tool in a diverse range of family medicine and pediatric care settings. Our primary objectives were to examine the uptake of screening for poverty, evaluate its acceptability to patients, and explore HCPs’ experiences with implementation of the tool.

## Methods

This was an exploratory study of implementing a clinical poverty tool in a range of family medicine and pediatric care settings in the southeastern region of Ontario. The study was approved by the Queen’s University Health Sciences and Affiliated Teaching Hospitals Research Ethics Board and all participants provided consent to participate and to publish the findings.

### HCP recruitment and training

A convenience sample of HCPs was recruited from family medicine and generalist and specialist pediatrics. The family physicians and nurse practitioners were recruited from three models of primary care delivery and payment including family health teams (modified capitation physician payment, interdisciplinary team based model), community health centres (salaried physicians, interdisciplinary team model with a focus on serving vulnerable populations), and fee for service practices. The pediatricians were recruited from the Department of Pediatrics at Queen’s University. All participating HCPs attended a three-hour “Treating Poverty” workshop accredited by the College of Family Physicians of Canada. In addition to introducing the clinical poverty tool, the workshop examined definitions of poverty in Canada, the impact of poverty on health, and resources aimed at increasing financial support for people living in poverty. The workshop also described locally available resources to help low-income residents and a package with more detailed information on those resources was distributed to the HCPs.

### Implementation of clinical poverty tool and quantitative data collection and analysis

Participants were asked to implement Ontario’s clinical poverty tool during a three-month period (May 30, 2016 to August 26, 2016). Specifically, the HCPs were asked a) to screen all their patients for poverty; and b) to intervene in the event of a positive screen by asking a series of follow-up questions embedded in the clinical poverty tool (e.g., “Have you filled out and sent in your tax forms?”) and by referring patients to the resources covered in the “Treating Poverty” workshop. Consistent with what was taught in the workshop and the “real world” implementation plan, however—and recognizing that a “one size fits all” approach would not be feasible given the diversity of care settings—HCPs were not provided with detailed implementation instructions (e.g., at what point in the clinical encounter to screen patients), but were instead advised to tailor the approach to their setting.

Using a form developed for this study, HCPs were asked to manually track on a daily basis the number of patients (or adult caregivers for pediatric patients) screened, whether they screened positive for poverty, and if so, the type of intervention provided. The forms were stored by administrative staff at each site and were collected periodically by a research assistant during her site visits. At the end of the implementation period, administrative staff at each site provided the research team with the total number of patient encounters for each of the participating HCPs. The research team calculated a) the proportion of patients screened by dividing the self-reported number of patients screened by the total number of patient encounters; b) the proportion of patients who screened positive by dividing the number who screened positive by the number who were screened for poverty (both self-reported); and c) the proportion of patients who received some form of intervention by dividing the number of patients provided with an intervention by the number of patients who screened positive for poverty (again, both self-reported).

**Fig. 1:**
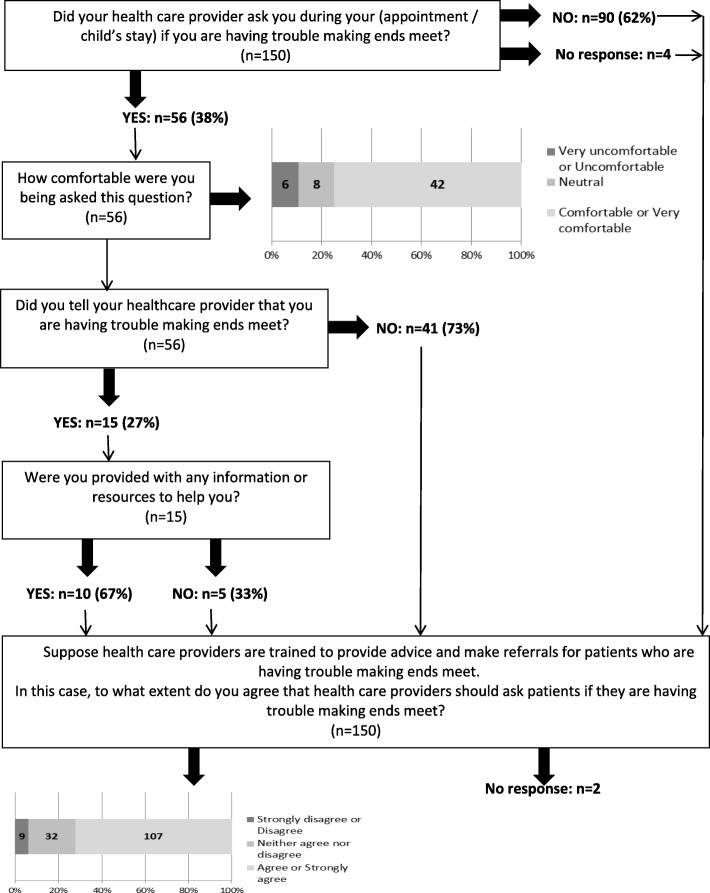
Patient survey questions and responses

A five-item questionnaire was developed to gauge patients’ comfort level with being screened for poverty and their views on whether HCPs should screen for and intervene on poverty (see text boxes in Fig. 1; note that not every question was applicable to every respondent). At the inpatient sites, if children had been under a participating HCP’s care, the unit clerks were asked to distribute the questionnaire to the parents when those children were ready to be discharged. Respondents were instructed to leave completed surveys in locked dropboxes that were located on the unit clerks’ desks.

A research assistant visited the outpatient sites on a periodic and rotating basis. The timing of these visits was decided in consultation with the participating HCPs to ensure that they were seeing patients during the scheduled times. On days when the research assistant was on site, reception staff were asked to distribute survey packages to the patients of participating HCPs when they checked in, and to invite them to complete the questionnaire after they had seen the doctor or nurse practitioner and to leave it with the research assistant. Patients were also advised that the research assistant could answer any questions about the study and could also help them complete the questionnaire, if so desired. The responses were entered into SPSS version 24.0 (IBM Corporation, Armonk, NY) and frequency distributions were run for each item on the questionnaire.

### Qualitative data collection and analysis

Following the implementation period, HCPs were invited to participate in a focus group. An interview guide (available on request) was developed to elicit HCPs’ views of how clinicians could address poverty, their perception of the appropriateness of screening for poverty, and their experience of implementing the tool. Questions relating to the latter topic included facilitators and barriers to screening and intervention and recommendations for improving the tool and integrating screening/intervention into routine care. An experienced facilitator was hired to run the focus groups. Discussions were transcribed verbatim.

Two family medicine residents—one of whom was a member of the research team (CK) —performed a thematic analysis using a phenomenological approach [24] to better understand the experience of HCPs with the implementation of poverty screening. Each resident reviewed the transcripts in their entirety and coded them using NVivo version 11.3.2 (QSR International, Burlington, MA) under the supervision of one of the co-investigators with a background in qualitative methods (EP). Following initial coding, the residents collaboratively reviewed their coding to develop consensus around emerging themes [24]. These were then summarized and illustrated with quotes.

## Results

### Quantitative findings

Twenty-two HCPs participated in the study, including nine family physicians, ten pediatricians, and three nurse practitioners, representing 12 clinical settings (Table 1).

**Table 1:** Implementation sites for clinical poverty tool

Setting	Sites	Scope of care	Patient population	Number of providers recruited
Community Health Centre^a^	1 urban site 1 site targeting addictions and homeless adults 1 rural site	General family medicine, targeted addictions care	All, focus on patients with low socioeconomic status	5 physicians 2 nurse practitioners
Family Health Team^b^	1 academic family health team 1 non-academic family health team	General family medicine	All	3 physicians 1 nurse practitioner
Family medicine	1 private fee-for-service practice with focus on vulnerable populations	Prenatal care, addictions medicine	Pregnant patients, patients experiencing addictions	1 physician
Pediatric inpatient	3 pediatric inpatient units 1 neonatal intensive care unit	General inpatient pediatrics, critical care pediatrics, well newborn service, neonatal intensive care	Well newborns, ill newborns and children up to 18 years of age	4 physicians (3 overlapping with outpatient pediatrics)
Pediatric outpatient	1 children’s outpatient centre 1 developmental clinic	General pediatrics, specialty pediatrics (genetics, respirology, neurology, infectious disease), urgent care pediatrics, developmental pediatrics	Infants and children up to 18 years of age	9 physicians (3 overlapping with inpatient pediatrics)

^a^ Community Health Centres are community-governed primary health care organizations staffed by interdisciplinary teams. They focus on health promotion and community development programs to reduce the negative impacts of social and environmental factors on health

^b^ Family Health Teams consist of doctors, nurses, nurse practitioners, social workers, dietitians, and other health care professionals who work collaboratively to deliver patient-centred care in Ontario

There were 6364 patient encounters and HCPs reported screening 581 patients (9%) over the three-month implementation period (Table 2). Among those patients, 165 (28%) screened positive for poverty. The majority of patients who screened positive (*n* = 122, 74%) were provided with an intervention, including referral to internal resources, particularly social work (available at all sites except the private fee-for-service practice), referral to a community agency, advice to file tax returns, referral to a locally developed website identifying community resources, and referral to a community support worker (available only to clients of the family health teams and community health centres).

**Table 2:** Screening and intervention proportions

Setting/Site	Patient encounters during implementation period^a^ n	Patients screened^b^ n (%)	Patients who screened positive^b,c^ n (%)	Patients provided with intervention^b,d^ n (%)
Community Health Centre	1831	124 (6.8)	48 (38.7)	39 (81.3)
Family Health Teams and private fee-for-service family medicine practice	1606	189 (11.8)	59 (31.2)	37 (62.7)
Pediatric inpatient	263^e^	28 (10.6)	3 (10.7)	3 (100)
Pediatric outpatient	2664	240 (9.0)	55 (22.9)	43 (78.2)
Total	6364	581 (9.1)	165 (28.4)	122 (73.9)

^a^ Based on billing remittances, electronic medical records, or manual tally of patients who were under the care of at least one of the participating health care providers during the implementation period (inpatient sites only). For outpatient sites, indicates the total number of encounters (except for one family practice, which only provided the total number of unique patient encounters)

^b^ Self-reported by participating health care providers

^c^ Using number of patients screened as the denominator for calculating proportions

^d^ Using number of patients who screened positive as the denominator for calculating proportions

^e^ Number of admissions during the implementation period where the patient was under the care of at least one participating health care provider

There were 150 respondents to the patient questionnaire, 52% of whom were seen by a pediatrician and 48% by a family physician or nurse practitioner. The majority of respondents felt comfortable or very comfortable being asked by their HCP if they were having trouble making ends meet (42/56, 75%) and agreed or strongly agreed that HCPs should screen their patients for poverty (107/148, 72%) (Fig. 1).

### Qualitative findings

Eighteen HCPs (82%) attended one of five semi-structured focus groups of mixed composition lasting approximately one hour. Five major themes emerged (selected quotes to illustrate each theme are shown in Table 3).

**Table 3:** Themes that emerged from focus group discussions around implementing a clinical poverty tool and select participant quotes

Theme	Quote
Health care providers recognize the importance and value of screening all patients for poverty	People who responded “yes I have trouble making ends meet” were people I wouldn’t expect. (FG3, neonatologist) Someone living in poverty is going to be at much higher risk of a whole bunch of things that I’m responsible for than someone who isn’t. (FG4, Family physician) Was a humbling experience (…) found out stuff about people that I had been seeing for five years. (FG3, NP) Just because our patients have an adequate income range doesn’t mean they’re not having a hard time making ends meet. (FG5, NP) People who replied yes I have trouble making ends meet were people that I wouldn’t expect (FG3, Nronatologist) All of us who participated in the project understand the very real implications of living in poverty, and how that affects people’s health. (FG5,Family physician)
Individual, practitioner-level barriers to screening	I was shocked at how difficult it was to remember to ask it. For me it was to get into a new habit. (FG2, Family physician) When I was seeing a brand new consult (…) I would remember to ask them because it would come up naturally to a degree. (FG3, Pediatrician)
Systemic and organizational barriers to screening	If I had some way of making a notation in the EMR (…) then it would get done. (FG4, Family physician) It is not a question you can just ask and quickly move on to the rest of the clinical interview (…) it does take time, and sometimes that’s a barrier. (FG5, Family physician)
Contextual factors impacting screening	Family practice is the better place, I think. Because I have no specific agenda with anybody (…) I can just carry something on if I need to. (FG4, Family physician) It was really awkward to ask someone who you’d known for five or six years! (FG3, NP)
HCPs’ lack of expertise and lack of effective interventions and available resources to offer patients who screen positive	I felt uncomfortable. I don’t think it was a bad thing to do, I just didn’t know quite how I would get over that. (FG4, Neonatologist) I would be terrified they’d screen positive, because, what would I do? (FG4, Neonatologist) And I think that even if we have a reasonable facility with some of the more common resources, people's individual needs vary, and I think it merits somebody who can do a deeper dive. (FG5, Family physician) I don’t think any of us have the adequate resources. There aren’t adequate resources. And that’s really hard. (FG3, NP) The reality is, I have a lot of people who are connected with everything under the sun, and it’s still not enough to live on for a month. (FG4, Family physician)

#### Theme 1: support for the importance and value of screening all patients for poverty

HCPs thought that poverty is relevant to clinical care and worth discussing with their patients. Some providers reported surprise at uncovering “hidden poverty”, even among some patients they had treated for a number of years, which reinforced their support for universal screening. They noted that patients experience fluctuations in their socioeconomic circumstances, and therefore thought that poverty screening should not be a one-time event in the course of the provider-patient relationship. Suggestions in this regard included screening at times of the year when expenses are typically higher (e.g., winter, start of the school year) and at certain life stages or events (e.g., pregnancy, major illness, job loss).

#### Theme 2: individual, practitioner-level barriers

Multiple personal barriers to universal screening were identified. These included challenges with remembering to perform a new task; uncertainty around how to integrate screening into the clinical encounter; and feelings of awkwardness around asking patients about their financial situation. This awkwardness was particularly felt when screening patients whom the provider had known for years, though it was in these instances that the hidden poverty mentioned above was sometimes identified.

#### Theme 3: systemic and organizational barriers

Participants also identified systemic and organizational barriers to implementation. Some were related to the specific health care organization, for instance characteristics of an electronic medical record which made remembering to screen or recording the results of poverty screening more or less difficult, as well as the structure and length of appointments. Challenges attributed to the organization of the healthcare system as a whole included the number of other screening tasks expected of HCPs at a given primary care appointment.

#### Theme 4: contextual factors

The tool was viewed as more difficult to implement in inpatient units where multiple providers overlapped, and in urgent care settings where providers felt there were more pressing biomedical issues to prioritize. Providers felt that primary care and other settings where ongoing provider-patient relationships were developed were more conducive to screening.

#### Theme 5: HCPs’ lack of expertise, and inadequate effective interventions and resources to offer patients who screen positive

This theme was succinctly captured in the words of one provider: *“I would be terrified they’d screen positive, because, what would I do?”* Many HCPs did not feel confident in their knowledge of community and financial resources for those living in poverty. However, even those who felt confident, or had access to more comprehensive organizational resources (Community Health Centre sites, for example), noted that adequate resources are simply not available in many cases to meet their patients’ needs. The combination of a sense of personal lack of competence with respect to training and knowledge, and a lack of adequate solutions were perceived as barriers to implementing the tool.

## Discussion

This exploratory study of the implementation in primary and pediatric care settings of a clinical poverty tool that recommends universal screening revealed low screening rates (9% according to self-reported numbers) among a group of early adopters actively committed to the process. Most patients viewed poverty screening as acceptable and desirable and the majority who screened positive were offered assistance. Participating HCPs identified poverty screening as important but identified multiple barriers to successful implementation, including systemic and logistic factors, as well as HCPs’ discomfort, lack of confidence, and lack of available resources to support their patients.

This work should be interpreted in the context of an increasing body of literature describing implementation of screening and interventions to address unmet social needs in health care settings. In the U.K., embedding staff in general practices to provide advice on accessing welfare benefits has demonstrated increased income, though not improvements in health outcomes [25–28]. In the U.S., routine collection of social needs data is now recommended by the National Academy of Medicine [29] and the Centers for Medicare and Medicaid Innovation [30], and has been implemented in Health Leads [31] and the National Association of Community Health Centers [32]. In the latter case, significant attention has been paid to developing workflows and data plans. Health centers that implemented social needs screening reported that screening was very rarely carried out by physicians, but was largely supported by non-clinical and non-physician clinical staff [33, 34].

Health care is provincially funded in Canada and there are no Ontario-wide structural mechanisms that support poverty screening in the province. The St. Michael’s Family Health Team in Toronto, Ontario has been a leader in developing the clinical poverty tool, implementing routine collection of sociodemographic data and using this data to apply an equity lens to clinical care [35–38]. Ontario’s Community Health Centres have had a focus on addressing health inequities since their inception [39]. However, to our knowledge there have been no reports quantifying measures related to the implementation of poverty screening and intervention at these centres or in other parts of Canada.

Our findings and the existing literature suggest three key issues need to be addressed to ensure more successful and wide-spread implementation of the clinical poverty tool. First, organizational engagement is paramount. Browne et al. [40] describe a framework to enhance the capacity of primary health care organizations to provide “equity-oriented care” and articulate the need for organizational buy-in when intervening in equity-related areas such as poverty. A multidisciplinary and organizational approach with high-level system planning is needed. Additionally, changing entrenched behaviours is challenging even among those committed to change. While audit and feedback and point of care computer reminders have been shown to effect changes in HCP behaviour [41, 42], the changes were small, and the intensity of training required was more than the three-hour workshop provided in this study. Additionally, while it is unclear which clinical staff are best placed to screen and to intervene in poverty, some studies would support implementation by non-physician staff [33, 34].

Second, HCPs identified concerns about feeling inadequately prepared for the complexities raised by these topics. Feelings of powerlessness, lack of control, and lack of effective interventions have been identified as limitations to HCP engagement [43, 44]. Integrating poverty screening, social determinants of health, and other equity and advocacy-related topics into undergraduate and postgraduate medical training programs is crucial to effect a cultural shift and to ensure the sustainability of clinical equity interventions [45–47].

Finally, there are gaps in services available to support patients living in poverty. Health inequities are, by definition, socially constructed and unjust [40, 48], and HCPs noted that there are insufficient resources for their patients to address the complexities of poverty. Some healthcare organizations have established additional local resources. Recently, Ontario’s South East Local Health Integration Network began testing a program of community support workers for family health teams. Community support workers act as system navigators, assisting patients in everything from income supports to housing to completion of government forms and application for insurance funding. While HCPs have identified this resource as helpful there are insufficient workers to support the population needs, a situation that could be further exacerbated should poverty screening become more broadly implemented. However, even a competent worker cannot find adequate resources when none are available, suggesting the need for broader advocacy around the social determinants of health.

### Strengths and limitations

The breadth of evaluation sites and practitioner types were strengths of this study. Participation in the focus groups was high.

Our study also had several limitations. As previously noted, this was meant to be a “real world” implementation without reminders to screen and intervene. Nevertheless, the study coordinator acted as a prompt during site visits. The HCPs were predominantly primary care pediatricians, nurse practitioners, and family doctors, although they also included specialist pediatricians working in inpatient and specialized settings which are not broadly generalizable to primary care. While their input adds interesting information about challenges to screening in these environments, this was not the focus of the study. That said, the patients screened in inpatient settings represented only 4.8% of total patients screened, and while current Canadian recommendations do not extend to screening in inpatient settings, there are precedents for screening in inpatient pediatric settings elsewhere [49, 50].

The numerators used to calculate the screening and intervention proportions (and the denominator for the latter) were based on self-reported numbers and may be subject to error. This may explain the discrepancy between the proportions of patients screened for poverty based on numbers self-reported by the HCPs (9%) versus those derived from the patient survey (38%). However, it is also possible that patients who were screened may have been more likely to complete the survey. Regardless, the focus group discussions confirmed that screening proportions were low. The HCPs who participated were a self-selected group, they may have been more likely to be supportive of poverty screening. Patients were surveyed non-randomly, Both these limitations, may limit the generalizability of our findings. Nevertheless, these findings underscore the large gap between the ideal of universal poverty screening in clinical practice and the reality of how achievable that goal is without concerted efforts to support implementation efforts.

## Conclusion

Low screening rates were observed among clinicians implementing a clinical poverty tool, yet screening was largely viewed by patients and HCPs as acceptable and important. There is a pressing need for more research to improve implementation and to evaluate the effectiveness of clinical poverty tools in reducing poverty and improving health outcomes. Additional research is also needed to determine whether the focus should be exclusively on poverty or on multiple social determinants of health and the appropriate timing and frequency of screening, to effectively integrate screening into electronic health records, and to establish effective workflows for poverty screening and intervention.

## Data Availability

Data are available upon request by contacting the corresponding author. The full data are not freely available to respect the confidentiality of our participants, ensure data integrity, and avoid scientific overlap between projects.

## Abbreviation

HCP: Health Care Practitioner

## References

[1] Statistics Canada. Low income statistics by age, sex and economic family type, Canada, provinces and selected census metropolitan areas (CMAs) CANSIM Table 206–0041. http://www5.statcan.gc.ca/cansim/a26?lang=eng&retrLang=eng&id=2060041&pattern=&tabMode=dataTable&srchLan=-1&p1=-1&p2=9. Accessed July 14, 2016.

[2] Unicef. Building the Future: Children and the Sustainable Development Goals in Rich Countries. *Innocenti Report Card.* 2017;14:56–56.

[3] Mikkonen J. Raphael D. Social Determinants of Health: The Canadian Facts.

[4] Fletcher J, Wolfe B (2014). Increasing our understanding of the health-income gradient in children. Health Econ.

[5] Fitzpatrick T, Rosella LC, Calzavara A (2015). Looking beyond income and education. Am J Prev Med.

[6] Aber JL, Bennett NG, Conley DC, Li J (1997). The effects of poverty on child health and development. Annu Rev Public Health.

[7] Halfon Neal, Hochstein Miles (2002). Life Course Health Development: An Integrated Framework for Developing Health, Policy, and Research. Milbank Quarterly.

[8] Pascoe J. M., Wood D. L., Duffee J. H., Kuo A. (2016). Mediators and Adverse Effects of Child Poverty in the United States. PEDIATRICS.

[9] Hertzman Clyde (2010). Social Geography of Developmental Health in the Early Years. Healthcare Quarterly.

[10] Shonkoff JP, Garner AS (2011). Committee on psychosocial aspects of C, et al. the lifelong effects of early childhood adversity and toxic stress. Pediatrics..

[11] Collège L, Médecins D, Famille D, Canada D. Social determinants of health BEST ADVICE THE COLLEGE OF FAMILY PHYSICIANS OF CANADA BEST ADVICE – SOCIAL DETERMINANTS OF HEALTH. 2015.

[12] Canadian Medical A. Health Care in Canada: what makes us sick? 2013.

[13] Canadian Medical Association Submission on Motion 315 (Income Inequality). 2013.

[14] Kralj B (2012). Kantarevic, J. Primary care in Ontario: reforms, investments and achievements.

[15] Guttman A. SS, Jaakkimainen L. . In: Jaakkimainen L. UR, Klein-Geltink JE., Leong A., Maaten S., Schultz SE., Wang L. , ed. *Primary Care in Ontario: ICES Atlas.* Toronto: Institute for Clinical Evaluative Sciences.

[16] Poverty: A Clinical Tool for Primary Care Providers | Programs and Practice Support | CPD | The College of Family Physicians Canada. In.

[17] Ontario College of Family Physicians. https://thewellhealth.ca/wp-content/uploads/2016/07/Poverty_flow-Tool-May1.pdf.

[18] Brcic Vanessa, Eberdt Caroline, Kaczorowski Janusz (2011). Development of a Tool to Identify Poverty in a Family Practice Setting: A Pilot Study. International Journal of Family Medicine.

[19] Brcic V, Eberdt C, Kaczorowski J (2015). Corrigendum to “development of a tool to identify poverty in a family practice setting: a pilot study”. International Journal of Family Medicine.

[20] Rourke DL, Rourke, J. . Rourke Baby Record: Evidence-Based Infant/Child Health Maintenance. 2017; www.rourkebabyrecord.ca

[21] Greig AA, Constantin, E., LeBlanc, C.MA., Riverin, B., Tak Sam Li, P., Cummings, C. An update to the Greig Health Record: Preventive health care visits for children and adolescents aged 6 to 17 years – Technical report. 2016; https://www.cps.ca/en/documents/position/greig-health-record-technical-report.

[22] Dosani N. Screening for poverty: identifying an important social determinant of health. Healthy Debate. 2012.

[23] Statistics Canada. Primary health care providers. 2016; https://www150.statcan.gc.ca/n1/pub/82-625-x/2017001/article/54863-eng.htm.

[24] Creswell JWP, Cheryl N. Qualitative inquiry and research design. California, USA: SAGE Publications; 2018.

[25] Adams J, White M, Moffatt S, Howel D, Mackintosh J (2006). A systematic review of the health, social and financial impacts of welfare rights advice delivered in healthcare settings. BMC Public Health.

[26] Abbott S (2002). Prescribing welfare benefits advice in primary care: is it a health intervention, and if so, what sort?. J Public Health.

[27] Paris JA, Player D (1993). Citizens' advice in general practice. BMJ (Clinical research ed).

[28] Mackintosh J, White M, Howel D (2006). Randomised controlled trial of welfare rights advice accessed via primary health care: pilot study. BMC Public Health.

[29] Medicine Io (2014). Capturing social and behavioral domains and measures in electronic health records.

[30] Alley DE, Asomugha CN, Conway PH, Sanghavi DM (2016). Accountable health communities — addressing social needs through Medicare and Medicaid. N Engl J Med.

[31] Onie RD, Lavizzo-Mourey R, Lee TH, Marks JS, Perla RJ (2018). Integrating social needs into health care: a twenty-year case study of adaptation and diffusion. Health Aff.

[32] PRAPARE. Implementation and action toolkit - NACHC. In. .

[33] Byhoff E, Cohen AJ, Hamati MC, Tatko J, Davis MM, Tipirneni R (2017). Screening for social determinants of health in Michigan health centers. The Journal of the American Board of Family Medicine.

[34] Gold RPMPH, Cottrell EPMPP, Bunce AMA (2017). Developing electronic health record (EHR) strategies related to health center Patients' social determinants of health. J Am Board Fam Med.

[35] Jones Marcella K, Bloch Gary, Pinto Andrew D (2017). A novel income security intervention to address poverty in a primary care setting: a retrospective chart review. BMJ Open.

[36] Aery Anjana, Rucchetto Anne, Singer Alexander, Halas Gayle, Bloch Gary, Goel Ritika, Raza Danyaal, Upshur Ross E G, Bellaire Jackie, Katz Alan, Pinto Andrew David (2017). Implementation and impact of an online tool used in primary care to improve access to financial benefits for patients: a study protocol. BMJ Open.

[37] Pinto AD, Glattstein-Young G, Mohamed A, Bloch G, Leung F-H, Glazier RH (2016). Building a foundation to reduce health inequities: routine collection of Sociodemographic data in primary care. J Am Board Fam Med.

[38] Kiran Tara, Pinto Andrew D (2016). Swimming ‘upstream’ to tackle the social determinants of health. BMJ Quality & Safety.

[39] Rayner J, Bayoumi I, Muldoon L, McMurchy D, Tharao W. Delivering Primary Health Care as Envisioned - A Model of Health and Wellbeing Guiding Community-Governed Primary Care Organizations. Journal of Integrated Care. 2018. 10.1108/JICA-02-2018-0014 PMC6091657 30166944

[40] Browne AJ, Varcoe C, Ford-Gilboe M, Wathen CN. Equip research team obotER. EQUIP Healthcare: An overview of a multi-component intervention to enhance equity-oriented care in primary health care settings *Intern*. 2015;14:152. 10.1186/s12939-015-0271-y PMC4688920 26694168

[41] Shojania KG, Jennings A, Mayhew A, Ramsay CR, Eccles MP, Grimshaw J. The effects of on-screen, point of care computer reminders on process and outcomes of care. *Cochrane Database of Systematic Reviews.* 2009(3):Art. No.: CD001096. 10.1002/14651858.CD001096.pub2 PMC4171964 19588323

[42] Ivers N, Jamtvedt G, Flottorp S, et al. Audit and feedback: effects on professional practice and healthcare outcomes. Cochrane Database Syst Rev. 2012. 10.1002/14651858.CD000259.pub3 PMC11338587 22696318

[43] Clements S, Cummings S (1991). Helplessness and powerlessness: caring for clients in pain. Holist Nurs Pract.

[44] Gutmanis I, Beynon C, Tutty L, Wathen CN, MacMillan HL (2007). Factors influencing identification of and response to intimate partner violence: a survey of physicians and nurses. BMC Public Health.

[45] Klein MD, Kahn RS, Baker RC, Fink EE, Parrish DS, White DC (2011). Training in social determinants of health in primary care: does it change resident behavior?. Acad Pediatr.

[46] Sharma M, Pinto AD, Kumagai AK (2018). Teaching the social determinants of health: a path to equity or a road to nowhere?. Acad Med.

[47] Colvin JD, Bettenhausen JL, Anderson-Carpenter KD (2016). Multiple behavior change intervention to improve detection of unmet social needs and resulting resource referrals. Acad Pediatr.

[48] Browne AJ, Varcoe CM, Wong ST (2012). Closing the health equity gap: evidence-based strategies for primary health care organizations. Intern..

[49] Berman RS, Patel MR, Belamarich PF, Gross RS (2018). Screening for poverty and poverty-related social determinants of health. Pediatr Rev.

[50] Zheng Daniel J., Shyr Derek, Ma Clement, Muriel Anna C., Wolfe Joanne, Bona Kira (2018). Feasibility of systematic poverty screening in a pediatric oncology referral center. Pediatric Blood & Cancer.

